# Mechanism of Action of Dengzhan Shengmai in Regulating Stroke from an Inflammatory Perspective: A Preliminary Analysis of Network Pharmacology

**DOI:** 10.1155/2021/6138854

**Published:** 2021-10-31

**Authors:** Yiqi Yan, Chao Sun, Xiaoting Rong, Rui Han, Shan Zhu, Rui Su, Ya Jin, Lin Li, Jun Liu

**Affiliations:** ^1^Laboratory of Pharmacology of Traditional Chinese Medicine Formulae Co-Constructed By the Province-Ministry, Tianjin University of Traditional Chinese Medicine, Tianjin 301617, China; ^2^State Key Laboratory of Component Traditional Chinese Medicine, Tianjin University of Traditional Chinese Medicine, Tianjin 301617, China; ^3^Graduate School, Tianjin University of Traditional Chinese Medicine, Tianjin 301617, China; ^4^Department of Radiology, Tianjin Union Medical Center, Tianjin 300121, China; ^5^Tianjin Central Hospital of Gynecology Obstetrics, Tianjin 300121, China

## Abstract

Stroke is a complicated disease with an increasing incidence and a very high mortality rate. A classical Chinese herbal medicine, Dengzhan Shengmai (DZSM), has shown to have therapeutic effects on stroke; however, its chemical basis and molecular mechanism are still unclear. In this study, a systems biology approach was applicable to elucidate the underlying mechanism of action of DZSM on stroke. All the compounds were obtained from databases, and pendant-related targets were obtained from various data platforms, including the TCM Systematic Pharmacology (TCMSP) database, TCM Integrated Database (TCMIP), High Throughput Experimental Reference Database (HERB), Comparative Toxicogenomics Database (CTD), SwissTargetPredicition, and SymMap, The Human Gene Database (GENECARD) and Comparative Toxicogenomics Database (CTD) were used for stroke disease target data, followed by network pharmacology analysis to predict the potential effect of DZSM on stroke. Animal experiments were intended to validate the underlying mechanisms. A total of 846 chemical components were compiled for the targets of DZSM drug, and quercetin, kaempferol, and Wuweizisu C are the highest chemical components compiled from DZSM. Overlapping with 375 disease-specific targets and 149 core targets, the core targets include TNF, IL-6, ALB, and AKT1, which are shown to regulate the disease process from an anti-inflammatory perspective. 198 enrichment messages were obtained by KEGG enrichment analysis, and we believe that the role of the AGE-RAGE signaling pathway in diabetic complications, TNF signaling pathway, and IL-17 signaling pathway is more important. Based on rat experiments, we also demonstrated that DZSM could effectively modulate the inflammation level of brain infarct tissues and effectively alleviate behavioral characteristics. Grouped together, our study suggests that the combination of network pharmacology prediction and experimental validation can provide a useful tool to describe the molecular mechanisms of DZSM in Chinese medicine (TCM).

## 1. Introduction

Stroke is a syndrome of localized cerebral arterial vascular blockage, resulting in hypoxia and ischemia of brain tissue in the area of vascular innervation, causing corresponding neurological dysfunction, which is the main cause of long-term disability and reduced quality of life in later life [1, 2]. Some studies have shown that ischemic stroke patients have varying degrees of spontaneous recovery of neurological function over time, which is dominant with appropriate rehabilitation and drug therapy [3–7]. It is generally believed that the inflammatory response is an important cause of neural remodeling, mainly including vascular regeneration, axonal sprouting, and synaptic remodeling [8–11], and inflammation levels can affect axon generation, while axonal sprouting and synaptic remodeling can promote the establishment of neural circuits and compensate for the innervation of damaged areas; therefore, regulation of inflammation levels is crucial to promote neural remodeling in stroke [12, 13]. Therefore, regulation of inflammation levels is critical to promote neurological remodeling after stroke.

Chinese medicine has been extensively used as an alternative therapy for stroke in China [14], in which proprietary Chinese medicine has the advantages of multitarget, multipathway, and multilinkage. Ginseng is the most commonly used Chinese patent medicine for the treatment of stroke, which consists of Radix et Rhizoma Ginseng, Radix et Rhizoma Ginseng, Radix et Rhizoma Macrocephala, and Radix et Rhizoma Wu Wei Zi and has the effect of benefiting Qi and nourishing Yin, invigorating blood, and strengthening the brain. It is utilized to treat the symptoms of stroke by removing blood stasis and with ginseng, maitong, and wu wei zi to form the famous formula sang wei sheng to benefit qi and nourish yin. Benefiting qi and nourishing yin means that when qi is healthy, blood moves and when yin is sufficient, the brain and kidney are combined, so that brain nerve function is normal and nerve function is improved and restored after stroke.

Modern pharmacological studies have shown that *Erigeron breviscapus* has anti-inflammatory, antioxidant, anticoagulant, and vascular protective effects [15, 16]. Clinical practice has confirmed that DZSM capsules and its active ingredients can effectively suppress the level of inflammation after ischemic stroke and contribute to neurological recovery and cognitive function improvement in patients [17–20]. Recent studies have found that the active ingredients of DZSM capsules can treat chronic brain tissue ischemia and hypoxia leading to neurosynaptic changes by regulating the levels of inflammatory factors [21]. Therefore, it is of major clinical significance to clarify the mechanism of DZSM on ischemic stroke.

## 2. Materials and Methods

### 2.1. Screening of Active Ingredients

The HERB database, Swiss database, TCMSP database, CTD database, and SYMMAP database were utilized to predict the ingredients of DZSM.

### 2.2. Prediction of Drug-Related Targets

The TCMSP, HERB SWISSTARGET, CTD, and SYMMAP database were used to predict the targets of DZSM, and then, the targets were predicted in the Uniport online protein database (Uniprot), to find the corresponding standard gene name, so as to obtain the related target of active ingredients of DZSM.

### 2.3. Prediction of Disease-Related Targets

Using the GENECARDS database and CTD database to obtain the relevant content of stroke and delete the repetitive target gene, we can obtain the relevant targets of stroke.

### 2.4. Finding Common Targets of Drugs and Diseases

By mapping the target information of DZSM with that of stroke, we can obtain the overlapped target Venn map and overlapped target information.

### 2.5. Construction of the Active Ingredient Target Network

The active components and overlapping genes of DZSM can be sorted out by using Cytoscape 3.7.1 software, and the network of active components and overlapping genes of DZSM can be drawn.

### 2.6. Construction of the Drug Disease Overlapping Target Protein Interaction Network (PPI)

The overlapping targets of DZSM and stroke were imported into the string data analysis platform for calculation, and PPI could be constructed according to the relationship between the targets.

### 2.7. Enrichment Analysis of the Kyoto Encyclopedia of Genes and Genomes (KEGG) Pathway

The overlapping target names of DZSM and stroke were transformed into Entrez gene ID by R language, and then, the KEGG-related information was obtained by analyzing and calculating with the KOBAS database. Then, the *P* value was used as the reference value to screen the related targets, and then, the R language was used to analyze the related content of pathway enrichment analysis.

### 2.8. Animals

Adult-specific pathogen-free SD rats (males, 250–300 g) were bought from the Experimental Animal Resource Center of Tianjin University of Traditional Chinese Medicine (TCM-LAEC2020058). They were housed at 20 ± 1°C, 40–60% humidity, with 12 h light/dark cycles. The procedure was carried out in accordance with the Guide for the Care of Laboratory Animals. The animal protocol was subject to approval by the Institutional Animal Care and Use Committee of Tianjin University of Traditional Chinese Medicine.

### 2.9. Experimental Groups

Rats were randomly divided into four groups: the control group, MCAO group, MCAO + low DZSM dose treatment group (113.4 mg/kg administered by gavage), and MCAO + high DZSM dose C group (226.8 mg/kg administered by gavage). Vehicle rats underwent the same procedure without carotid artery obstruction.

### 2.10. Focal Cerebral Ischemic Stroke Model

Rats were initially anesthetized with 5% isoflurane and then maintained with 1.5–2% isoflurane, 30% oxygen, and 70% nitrous oxide. The body temperature of the rats was maintained at 37°C (±1.0°C) using a thermostatic heating pad. After cutting the surface skin with a scalpel, the anterior soft tissue of the rat's neck was gradually and bluntly separated, and nylon thread was tied from the proximal and distal ends of the external carotid artery, with a dead knot at the distal end and a live knot at the proximal end, and the wall of the external carotid artery blood vessel was gently cut with vascular scissors, and a matching thread plug was inserted, and the thread plug was sequentially passed through the external carotid artery and the common carotid artery to the internal carotid artery, and the insertion of the thread plug was stopped when there was appropriate resistance to the thread plug, and the position of the Marker was observed and tied. When there was appropriate resistance to the wire plug, the insertion of the wire plug was stopped, the position of the Marker was observed, and the wire plug was tied to prevent it from falling off. The sham-operated group was modeled in the same way but without blocking the middle cerebral artery blood flow.

### 2.11. Model Evaluation

The stability of the model was evaluated by performing magnetic resonance scans on rats in the modeling and sham-operated groups that met the neurological function criteria 24 hours after the MCAO model was performed to determine the percentage of brain infarct area in rats. Rats were anesthetized with 10% chloral hydrate (preparation: 10 g dissolved in 100 mL saline) at a dose of 0.4 g/kg. After the rats were unconscious, they were fixed in a rat coil for scanning in the prone position while maintaining an anal temperature of 37 ± 0.5°C. Six rats in each group were randomly selected for magnetic resonance scanning. Magnetic resonance diffusion-weighted imaging (DWI) was used to detect acute cerebral infarction. Axial T2WI and DWI scans were performed, and the T2WI sequence was used to calculate the total area of the lesion, while the DWI sequence was used to calculate the area of the infarct core lesion, and the difference between the two was the area of the ischemic semidark zone.

Parameter settings: T2WI: repetition time 4300 ms, echo time 100 ms, layer thickness 1.5 mm, and matrix 70 × 70; DWI: repetition time 3800 ms, echo time 125 ms, layer thickness 1.5 mm, and matrix 80 × 80. Total area of lesion = total area of healthy brain tissue−area of normal brain tissue on the affected side, and outline the area of core lesion, area of ischemic semidark zone = total area of the lesion−area of the core lesion.

### 2.12. Physical Exercise

We recorded the weight change of the rats during the treatment period and conducted various behavioral evaluations of the rats' status in the balance beam experiment: a 2 m-long balance beam supported by an iron frame at each end (60 cm high), with a plastic cage at the end, was used as the end point of each experiment. Hanging wire experiment: a wire 50 cm long and 0.15 cm in diameter was fixed horizontally to a stand 37 cm above the ground, as described in our previous study, and the rats were placed in the middle of the wire rope and observed for 30 seconds each time, with the average score of 3 trials per animal recorded for each trial. Muscle strength experiments: tests were also performed at baseline, 3 and 7 days postoperatively. In a quiet environment, the rats were placed in a net with a mesh size of 2.3 cm × 2.3 cm, and the number of times the rats accidentally entered the net by their front paws within 2 minutes while walking in the net was counted as follows: (number of forepaw missteps measured on the contralateral side of the lesion−number of forepaw missteps on the ipsilateral side of the lesion)/total number of steps. Rats were needed to undergo acclimatization 3 days prior to modeling before operation.

### 2.13. Inflammatory Factor Assay

Inflammatory factor levels in infarcted tissue were measured using commercial ELISA kits at a 1 : 1 dilution (50 *μ*L) of each rat peri-infarct brain tissue. Intra- and interassay variation was <4–6% and <8–10%, respectively, with detectable concentrations ranging from 0.066–1024 ng/ml. Peri-infarct brain tissue concentrations of proinflammatory cytokines, including TNF-*α*, were measured with ELISA kits.

### 2.14. Statistical Analysis

Data are expressed as meaning ± SEM. Statistical analyses were performed using Graphical Board Prism version 4.0 software. Differences between groups were assessed by an unpaired *t*-test or ANOVA followed by Tukey's posttest. For correlation analysis, Pearson's correlation analysis and Spearman's correlation analysis served.

## 3. Results

### 3.1. Screening of Effective Components and Determination of Action Targets of DZSM

The TCMSP database has a total of 30 components of DZSM, of which 12 belong to ginseng, 10 to lampblackberry, and 8 to northern *Schisandra*. It is to be noted that these herbs have some components in common. All of them met the requirements of OB ≥ 40% and DL index ≥0.2. After eliminating the overlap, they were further analyzed as candidate bioactive components, and the details are shown in Table 1. Since the information was not included in the TCMSP database, a search using the TCMIP database yielded information on 35 related components. Among all the components, quercetin, kaempferol, and wuweizisu C were shown to be the three components with the highest degree values, respectively. Modern pharmacological studies have shown that both have anti-inflammatory effects.

DZSM compound indicators were obtained by searching the TCMSP and TCMIP online databases, combining the data of TCMSP, Herb, SwissTargetPrediction database, CTD, and SYMMAP and using the relevant target prediction techniques in each platform to screen the predicted targets of the abovementioned active ingredients and eliminate duplicate targets, finally obtaining a total of 744 herbal prediction targets.

**Figure 1:**
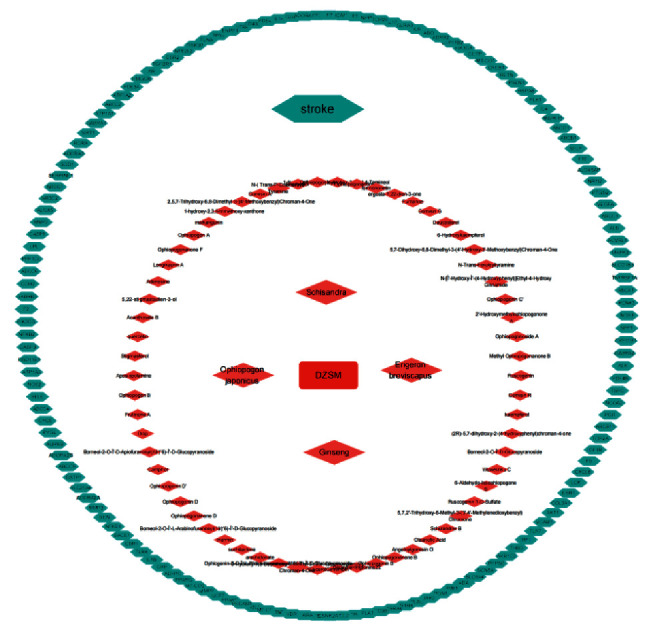
Chemical composition core target network diagram of DZSM. The red nodes represent the chemical components in DZSM, and the green nodes represent the core targets of the drug to treat the disease.

We searched the GENECARD, CTD, and TCMIP databases and screened for known target genes related to stroke. We combined target genes from each database, removed duplicate disease targets, and retrieved 372 disease targets. We combined 744 predicted herbal targets to obtain information on 91 specific targets for DZSM for stroke. We constructed a component-target network diagram based on the retrieved data, as shown in Figure 1.

### 3.2. PPI Network Construction

**Figure 2:**
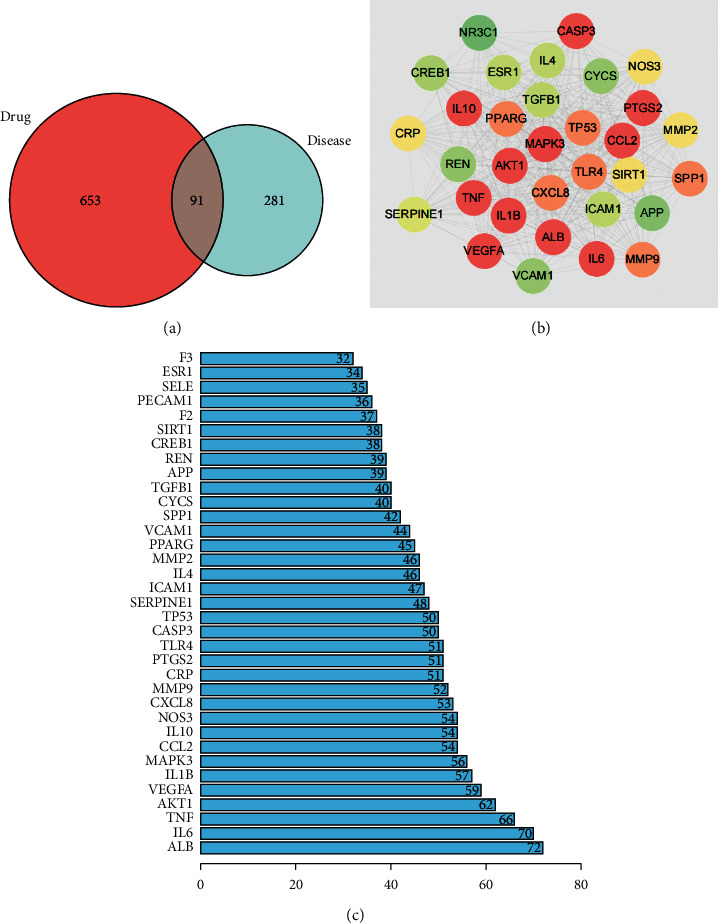
(a) Overlapping target Venn diagram, stroke and DZSM co-overlap; (b) PPI network analysis of the core target. The colors in the PPI network diagram represent degree statistical trends, from green to red, and degree values from low to high; and (c) the target degree values in the overlapping target PPI network were counted, and TOP 32 was plotted from the highest to lowest.

91 cross-target genes mentioned above were imported into the STRING online platform to obtain protein-protein interaction data columns, construct PPI network graphs, and analyze the data for topological heterogeneity. In order to more accurately understand the potential protein targets of DZSM-regulated stroke, the topology analysis data were screened, and targets with TOP32° values were selected as the core targets of DZSM-regulated diseases, and the topology data were imported into Cytoscape 3.7.1 to construct the core target network graph. As shown in Figure 2, the core targets of PPI are TNF,IL-6,ALB,AKT1,VEGFA, and CREB1, which play a key role in the regulation of stroke by DZSM (Figure 2).

### 3.3. Enrichment Analysis of the KEGG Pathway

In order to identify overlapping genes associated with stroke, we performed an enrichment analysis of the KEGG pathway to elucidate the associated signaling pathway. The *y*-axis represents the signaling pathway, and the *x*-axis indicates the number of genes enriched for the term. The redder the color, the smaller the p.adjusted value (FDR); it also indicates higher confidence and importance. Conversely, the more blue the color, the larger the value of p.adjust. The results suggest that the mechanism of action of DZSM in regulating stroke involves many signaling pathways.

**Figure 3:**
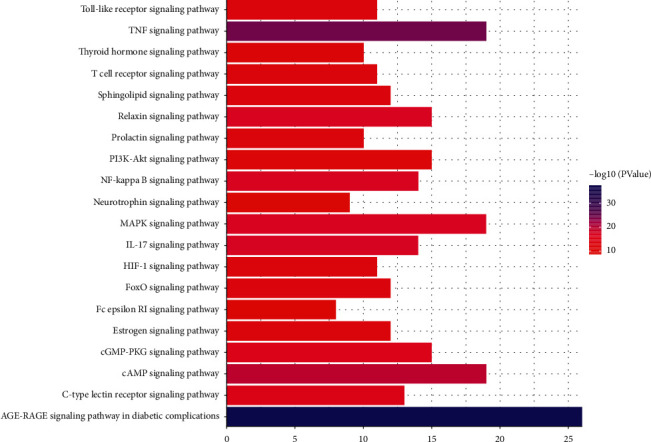
Enrichment analysis of the KEGG pathway. The colors in KEGG enrichment represent *P* value trends from red to blue with low to high correlation values.

Through enrichment analysis, we identified the functions of active ingredients in directly or indirectly regulating a number of pathways associated with the treatment of stroke, including TNF, cAMP, IL-17, and MAPK signaling pathways (Figure 3).

### 3.4. MCAO Model Stability Evaluation

Magnetic resonance scans show normal brain tissue and no lesions in the sham-operated rats. The percentage of the ischemic semidark zone area was (16.48 ± 1.29)% in the MCAO group, (16.18 ± 2.07)% in the low-dose group, and (15.00 ± 1.40)% in the high-dose group. The rats in the modeling group were significantly different from the rats in the sham-operated group (*P* < 0.001), and no significant differences were observed between the groups in the modeling group (*P* > 0.05).

### 3.5. DZSM Can Effectively Reverse the Physiological Changes Caused by MCAO

**Figure 4:**
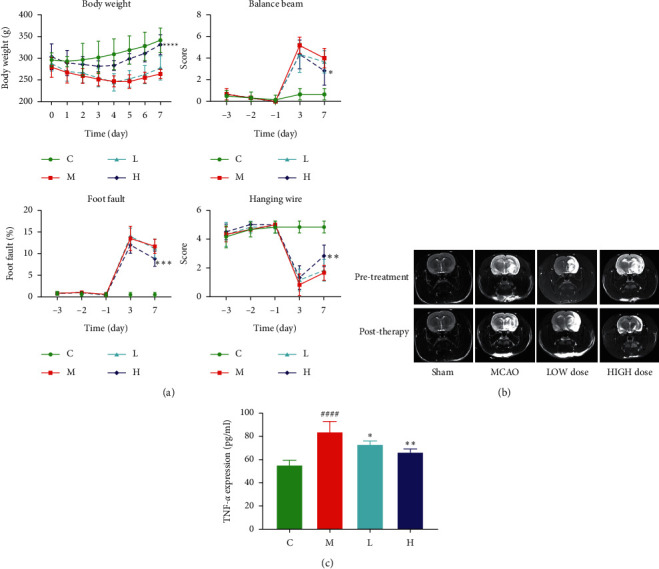
(a) Weight changes of rats in 7 days, balance beam experiment, foot-fault experiment, and hanging wire experiment; (b) model stability evaluation; and (c) detection of TNF-*α* in brain tissue by ELISA. Compared with the control group, *P* < 0.001; the low-dose group compared with the model group, *P* < 0.05; and the high-dose group compared with the model group, *P* < 0.01.

For correlation analysis, a total of 24 rats were subjected to MCAO. A decrease in body weight was found in the MCAO rats, compared to the normal group of animals. Also, with DZSM treatment, there were signs of weight reversion. Given the potent stimulating effects of DZSM on alleviating neurological deficits, DZSM may be involved in motor recovery. Rats in high dose of DZSM showed significantly better motor recovery, which was carried out at days 7 after MCAO. The results of the foot-fault test and hanging wire test showed that the motor abilities of rats were aggravated after surgery while improving following high dose of DZSM treatment (*P* ＜ 0.05) (Figure 4).

### 3.6. DZSM Modulates the Inflammatory Response Brought about by MCAO

Analysis of inflammatory factor levels assayed using DZSM administration in MCAO-modeled individuals reduced the levels of TNF-*α* in peri-infarct brain tissue, which indicates that the inflammatory response signal brought about by MCAO is inhibited by DZSM treatment. The inhibition of inflammatory factors from DZSM may slow down this process (Figure 4).

## 4. Discussion

Stroke is a complex disease, an acute cerebrovascular disease, and a group of diseases that cause damage to brain tissue due to sudden rupture of blood vessels in the brain or failure of blood to flow to the brain due to blockage of blood vessels [22] including ischemic and hemorrhagic strokes. It has become the first cause of death in China and the main cause of disability among Chinese adults. TCM consists of multiple compounds, and TCM may have a wide range of pharmacological and multitarget and pathway pharmacological activities that may be beneficial to the treatment of stroke [23]. On the other hand, this property of TCM may be, therefore, difficult to investigate the underlying mechanisms in depth. A network pharmacology approach that integrates systems biology and silicon techniques could provide a direction for the mechanistic study of complex TCM. In the current study, we used this approach to elucidate the pharmacological mechanism of stroke alleviation by DZSM.

Among the active ingredients of DZSM, the compounds with the highest number of connected targets are quercetin and kaempferol. Pharmacological studies have revealed that quercetin and kaempferol have been shown to exert neuroprotective effects in stroke patients through anti-ischemia, anti-free-radical oxidation, and inhibition of inflammatory responses [24–27].

The 91 targets acquired by combining the component-target protein PPI with the disease target protein PPI are the targets corresponding to the chemical components in DZSM and also the targets related to stroke, so these 91 targets are the core targets of DZSM for the treatment of stroke, and the targets interact with each other through 400 interactions to influence the treatment of the disease.

The core targets of DZSM for stroke are TNF, AKT1, VEGFA, and IL-1*β*, which are highly correlated. It has been demonstrated that TNF-*α* protein is abundantly expressed in brain tissue during ischemia and hypoxia and plays an important neurotoxic role in the pathogenesis and pathology of ischemic stroke by inducing the release of potent vasoactive substances, leading to vasoconstriction, reducing local cerebral blood flow, and increasing capillary permeability to promote the development of cerebral ischemia and edema [28]. VEGFA is a member of the VEGF family of vascular endothelial growth factors, which plays an important role in the process of angiogenesis after cerebral ischemia, and the administration of VEGFA to the MCAO rat model can increase neuroprotective effects. VEGFA can increase the density of biological microvessels in the ischemic semidark zone and promote angiogenesis after administration [29].

Based on enrichment analysis of the KEGG pathway in the core targets, it is clear that the AGE-RAGE signaling pathway, IL-17 signaling pathway. TNF signaling pathway, and cAMP signaling pathway are significantly enriched. Previous studies have shown that AGEs are irreversible end products formed by glycosylation of macromolecules, and one of their receptors, RAGE, belongs to the immunoglobulin superfamily [30]. In pathological states such as inflammatory response, ischemia-reperfusion injury, and hypoxia, the AGE-RAGE axis is activated [31]; on the one hand, the transcription factor NF-*κ*B is activated and AP-1 is inhibited, stimulating the activation of inflammatory response factors and coagulation factors leading to an expanded inflammatory response, increased endothelial cell damage, reduced synthesis of proangiogenic factors such as VEGF, and inhibition of vascularization; on the other hand, increased NF-*κ*B expression can upregulate RAGE, forming a positive feedback loop that further aggravates the abovementioned situation [32]. The pathophysiology of ischemic stroke is very complex, and the ensuing inflammatory and immune responses can aggravate the disruption of the blood-brain barrier and the development of brain edema, leading to secondary brain injury [33]. Astrocytes are the main source of IL-17, and the previous literature has shown that IL-17-mediated neurological responses exacerbate neurological injury after ischemic stroke, but recent studies have shown that IL 17A mediates cortical astrocytes, alleviates ischemic injury, and thus, affects neurological outcome in rats with ischemic stroke [34, 35]. cAMP is one of the important intracellular second messengers, which play a cerebral protective role by activating the PKA signaling pathway, mediating cAMP response element-binding protein CREB, and regulating the formation of neuronal regenerative synapses [36].

We made an MCAO rat model and tested it by neurological function score, and staining and motor function confirmed the successful model construction. Basing on this model, ischemia may extend to the entire vascular region of MCAO and lead to focal metabolic disturbances of the infarct, selective neuronal necrosis, and brain edema.

The Zea-Longa scale is the current standard for measuring neurological deficits in rats. We examined the locomotor behavior of rats in the MCAO group, sham-operated rats, and rats in different administrative groups using various behavioral methods. Consistent with previous findings, after focal cerebral ischemia, rats showed a significant decrease in balance beam time and a response of shortened suspension time, which was closely related to the size of the cerebral infarct and tended to occur in association after drug administration.

Given the effective stimulatory effect of DZSM in alleviating neurological deficits, DZSM may be involved in motor recovery. Rats with high doses of DZSM exhibited significant motor recovery, which was performed on day 7 after MCAO. The results of the balance beam, hanging wire test, and step-error test showed that the locomotor ability of the rats was aggravated after MCAO surgery and had a palliative effect after the administration of DZSM, where it improved after high-dose DZSM treatment. Also, inflammatory factors of cerebral infarct tissues were found to be significantly reduced and altered by DZSM treatment. This indicates that DZSM has a modulating effect on the improvement of the behavioral ability of rats after surgery.

Overall, here, the relationship and mechanism of DZSM as a multicomponent and multitarget drug interacting with stroke was systematically described using a network pharmacology approach. First, the relevant components, key targets, and enrichment pathways of DZSM were identified through a systematic analysis. These findings are valuable because understanding the biological functions of DZSM is important for the development of antistroke drugs, and our study provides insight into the application of TCM for the treatment of strong stroke, but the specific molecular biological mechanisms need further intensive study.

## Figures and Tables

**Table 1:** Chemical composition of Wuweiz, Renshen, and Dengzhanxixin selected from the TCMSP.

Mol ID	Molecule name	Drug	OB (%)	DL
MOL002879	Diop	Ginseng	43.59	0.39
MOL000449	Stigmasterol	*Ophiopogon japonicus*	43.83	0.76
MOL003648	Inermin	Ginseng	65.83	0.54
MOL000422	Kaempferol	Ginseng/*Erigeron breviscapus*	41.88	0.24
MOL005308	Aposiopolamine	Ginseng	66.65	0.22
MOL005314	Celabenzine	Ginseng	101.88	0.49
MOL005320	Arachidonate	Ginseng	45.57	0.2
MOL005321	Frutinone A	Ginseng	65.9	0.34
MOL005356	Girinimbin	Ginseng	61.22	0.31
MOL005360	Malkangunin	Ginseng	57.71	0.63
MOL005384	Suchilactone	Ginseng	57.52	0.56
MOL000787	Fumarine	Ginseng	59.26	0.83
MOL004624	Longikaurin A	*Schisandra*	47.72	0.53
MOL005317	Deoxyharringtonine	*Schisandra*	39.27	0.81
MOL008956	Angeloylgomisin O	*Schisandra*	31.97	0.85
MOL008957	Schizandrer B	*Schisandra*	30.71	0.83
MOL008968	Gomisin-A	*Schisandra*	30.69	0.78
MOL008974	Gomisin G	*Schisandra*	32.68	0.83
MOL008978	Gomisin R	*Schisandra*	34.84	0.86
MOL008992	Wuweizisu C	*Schisandra*	46.27	0.84
MOL000098	Quercetin	*Erigeron breviscapus*	46.43	0.28
MOL000392	Formononetin	*Erigeron breviscapus*	69.67	0.21
MOL000816	Ergosta-7,22-dien-3-one	*Erigeron breviscapus*	44.88	0.72
MOL001040	(2R)-5,7-dihydroxy-2-(4-hydroxyphenyl)chroman-4-one	*Erigeron breviscapus*	42.36	0.21
MOL002712	6-Hydroxykaempferol	*Erigeron breviscapus*	62.13	0.27
MOL002914	Eriodyctiol	*Erigeron breviscapus*	41.35	0.24
MOL005922	Acanthoside B	*Erigeron breviscapus*	43.35	0.77
MOL007963	1-Hydroxy-2,3,5-trimethoxy-xanthone	*Erigeron breviscapus*	101.06	0.3
MOL007984	Δ5,22-Stigmastadien-3-ol	*Erigeron breviscapus*	43.83	0.76

Prediction results of potential targets of DZSM in the treatment of stroke.

## Data Availability

The datasets used and/or analyzed during the current study are available from the corresponding author on reasonable request.
